# Novel Fusari- and Toti-like Viruses, with Probable Different Origins, in the Plant Pathogenic Oomycete *Globisporangium* *ultimum*

**DOI:** 10.3390/v13101931

**Published:** 2021-09-25

**Authors:** Miki Fukunishi, Shinsaku Sasai, Motoaki Tojo, Tomofumi Mochizuki

**Affiliations:** Graduate School of Life and Environmental Sciences, Osaka Prefecture University, Sakai 599-8531, Japan; goinghome-0114@outlook.jp (M.F.); sokatanmen@gmail.com (S.S.); tojo@plant.osakafu-u.ac.jp (M.T.)

**Keywords:** codon usage bias, convergent evolution, fusarivirus, *Globisporangium ultimum*, oomycete virus, *Pythium*, totivirus, transmission

## Abstract

To further classify the oomycete viruses that have been discovered in recent years, we investigated virus infection in the plant-parasitic oomycete *Globisporangium ultimum* in Japan. Double-stranded RNA detection, high-throughput sequencing, and RT-PCR revealed that the *G. ultimum* isolate UOP226 contained two viruses related to fusarivirus and totivirus, named Pythium ultimum RNA virus 1 (PuRV1) and Pythium ultimum RNA virus 2 (PuRV2), respectively. Phylogenetic analysis of the deduced amino acid sequence of the RNA-dependent RNA polymerase (RdRp) showed that fusari-like PuRV1 belonged to a different phylogenetic group than Plasmopara viticola lesion-associated fusari virus (PvlaFV) 1–3 from oomycete *Plasmopara viticola*. Codon usage bias of the PuRV1 RdRp gene was more similar to those of fungi than *Globisporangium* and *Phytophthora*, suggesting that the PuRV1 ancestor horizontally transmitted to *G. ultimum* ancestor from fungi. Phylogenetic analysis of the deduced amino acid sequence of the RdRp of toti-like PuRV2 showed a monophyletic group with the other toti-like oomycete viruses from *Globisporangium*, *Phytophthora,* and *Pl. viticola*. However, the nucleotide sequences of toti-like oomycete viruses were not so homologous, suggesting the possibility of convergent evolution of toti-like oomycete viruses.

## 1. Introduction

Oomycetes are fungal-like organisms classified as a member of the stramenopiles. Oomycetes include well-known plant pathogens *Pythium*, *Phytophthora*, and downy mildew, which cause serious losses in crop production worldwide [1]. Phylogenetic analyses have revealed that these pathogens appear to have radiated from a common ancestor and have evolved independently in three lineages of oomycete [2]. A long coevolution could have occurred between plant-parasitic oomycetes and their viruses, and it is possible to trace the phylogenetic relationship between oomycetes and viruses. Thus, the oomycete virus can be a good model for the coevolution of virus and host oomycete.

Recently, oomycete viruses have been reported to infect the plant parasites *Phytophthora*, *Pythium*, and downy mildew [3]. For example, Phytophthora infestans RNA viruses (PiRV)-1, -2, -3, and -4 were identified from the *Phytophthora infestans*, the causal agent of potato late blight [4,5,6,7]. High-throughput sequence analysis using total and small RNAs revealed 13 different bunya-like viruses and two toti-like viruses from a *Ph. condilina* single isolate in southern Portugal [8]. A variety of other viruses have been found in *Phytophthora* spp. [9,10,11,12,13,14]. Similar to *Phytophthora*, oomycete viruses were found in the *Pythium* and downy mildew [15,16,17,18,19,20,21]. We previously identified a novel partitivirus, toti-like viruses, and a bunya-like virus from *Globisporangium nunn*, *G. splendens*, and *G. polare* [22,23,24]. The genus *Globisporangium* complies with the oomycete species, with globose sporangia separated from genus *Pythium* [25]. Since this and our previous studies rely on earlier certified specimens identified before the genus name change, we include “Pythium” in the virus names. Interestingly, toti-like viruses have been detected in all three plant-parasitic oomycetes [8,12,13,21,23,24]. Toti-like oomycete viruses, Pythium polare RNA virus 1 (PpRV1), Pythium splendens RNA virus 1 (PsRV1), Phytophthora condilina RNA virus 1 (PcoRV1), Phytophthora cactorum virus 1 (PcRV1), and Plasmopara viticola lesion-associated toti-like virus (PvlaTLV) 1–2 are phylogenetically related based on molecular phylogenetic analysis using deduced amino acid sequences of the RdRp [8].

*Globisporangium*, *Pythium*, and *Phytophthora* are soil-borne plant pathogens with cosmopolitan distribution as well as soil fungal pathogens [26]. The oomycete and fungal pathogens in the phytobiome may interact because synergistic interactions have been reported in several combinations, e.g., [27]. An oomycete partitivirus found in a mycoparasitic *G. nunn* is highly similar to the fungal partitivirus [22]. However, viral transmission between fungi and oomycetes has not been described.

*Globisporangium ultimum* (Trow) Uzuhashi, Tojo and Kakish., formerly named *Pythium ultimum*, is one of the most important plant-pathogenic oomycetes that causes damping-off and root rot on >300 diverse hosts [1]. *G. ultimum* is one of the most pathologically and genomically characterized oomycetes because a whole-genome draft sequence was firstly determined in the genera *Globisporangium* and *Pythium* [28]. Using *G. ultimum*, we can study how viral infection affects the physiology and the virulence of the host *Globisporangium* at the gene expression level. However, no viruses infecting *G. ultimum* have been reported. Therefore, in this study, we searched for viruses that infect *G. ultimum*. We identified unclassified fusari- and toti-like viruses from *G. ultimum* isolates in Japan, which we named Pythium ultimum RNA virus 1 (PuRV1) and Pythium ultimum RNA virus 2 (PuRV2), respectively.

Molecular phylogenetic analysis of the deduced amino acid sequence of the RdRp domain showed that toti-like PuRV2 represents a monophyletic group with toti-like oomycete viruses PpRV1, PsRV1, PcoRV1, PcRV1, PvlaTLV1, and PvlaTLV2. In contrast, the fusari-like PuRV1 belongs to a different phylogenetic group from Plasmopara viticola lesion-associated fusariviruses (PvlaFVs) 1–3 from *Plasmopara viticola*, a grapevine downy mildew. The Codon Adaptation Index (CAI) and the Relative Codon Deoptimization Index (RCDI) analysis showed that the codon usage bias of the PuRV1 RdRp gene was more similar to those of fungi than *Globisporangium* and *Phytophthora*. We discuss the origin of the fusari-like virus and toti-like viruses of oomycetes.

## 2. Materials and Methods

### 2.1. Globisporangium ultimum Isolates

*Globisporangium ultimum* (Trow) Uzuhashi, Tojo and Kakish. isolates used in this study are shown in Table S1. The isolates were identified with ITS sequence and microscopic observations. Note that the UOP360 (MAFF242256), UOP386 (MAFF235799), and UOP388 (MAFF235801) have recently been re-identified as *Globisporangium* sp., *G.rostratifingens*, and *G. oryzicola*, respectively. The isolates were maintained on 10% (*v*/*v*) V8 agar (V8A) medium at 25 °C in the dark.

### 2.2. dsRNA Extraction

Mycelia used for nucleic acid extraction were propagated on 100 mL potato dextrose broth (PDB) liquid medium in an autoclaved Ziploc container (W156 × D117 × H53 mm, Asahi Kasei, Tokyo, Japan) at 25 °C for 7 days. After collecting mycelia and draining on paper towels, 2.5 g of mycelium was homogenized in liquid nitrogen. The powder was mixed with 5 mL extraction buffer (25 mM glycine, 0.1 M NaCl, 50 mM Tris-HCl pH 8.0, 1 mM EDTA pH 8.0, 1% SDS, 0.1% 2-Mercaptoethanol) and extracted with 5 mL phenol-chloroform twice. Total nucleic acid was precipitated with 0.3 M sodium acetate and ethanol. dsRNAs were purified from total nucleic acids using CF-11 cellulose following previously described methods [29] and by incubating with S1 nuclease (Promega, Madison, WI, USA) and DNase I (Promega) at 37 °C for 30 min.

### 2.3. High-Throughput Sequencing of dsRNA

High-throughput sequencing using MiSeq (Illumina, San Diego, CA, USA) was conducted following previously described methods [24]. A cDNA library for high-throughput sequencing was prepared using NEBNext Ultra RNA Library Kit for Illumina and Index primer set 1 (New England BioLabs, Ipswich, MA, USA) from 75 ng of dsRNA purified from UOP226. The library quality and quantity were assessed using an Agilent 4200 TapeStation with High sensitivity D1000 (Agilent Technologies, Santa Clara, CA, USA), and a Qubit 3.0 Fluorometer with a Quant-iT PicoGreen dsDNA Assay Kit (Life Technologies, Carlsbad, CA, USA), respectively. Sequencing was performed on a MiSeq benchtop sequencer using a MiSeq Reagent Kit Nano V2 for 2 × 150 paired-end (Illumina). The raw sequence reads were converted into a FASTQ data format, and then index and adaptor sequences were trimmed by Illumina Experiment Manager software (Illumina).

High-throughput sequencing using the DNBSEQ-G400 (MGI Tech, Shenzhen, China) was conducted by Bioengineering Lab. Co., Ltd., in Japan. Transcriptome analysis (200 bp paired-end sequencing) without rRNA removal was prepared from 93.3 ng of dsRNA newly purified from UOP226. The raw sequence reads were assembled by Velvet 1.2 [30] or rnaSPAdes with default parameter [31].

### 2.4. Northern Blot Analysis for dsRNA

Northern blot analysis for dsRNA was conducted following previously described methods [24]. DIG-labeled PCR probes were synthesized using the PCR DIG Probe Synthesis kit (Roche Diagnostics, Mannheim, Germany) with primer sets PuV-1-fw/-rv for contig 1 and PuV-4-fw/-rv for contig 4 (Table S2), and full-length cDNA from dsRNA as a template.

### 2.5. RT-PCR

The RT-PCR confirmed the presence of virus-like RNA with specific primer sets PuV-1-fw/-rv for contig 1 and PuV-4-fw/-rv for contig 4 (Table S2). The extracted total nucleic acids were used for the RT-PCR template. RT-PCR was performed using PrimeScript™ II High Fidelity One-Step RT-PCR Kit (Takara Bio, Shiga, Japan) according to manufacture instructions.

### 2.6. Determination of dsRNA Genome

The sequences of 5′- and 3′-ends of dsRNA were determined by RNA ligase-mediated-RACE (RLM-RACE) using a 5′-phosphorylated oligodeoxynucleotide primer and 5′ reverse and 3′ forward-specific primers (Table S2) following previously described methods [24]. For determination of 5′-end of PuRV1, PrimeScript™ II High Fidelity One-Step RT-PCR Kit (Takara Bio) with the adaptor primer (Tagged-primer) and PuRV1 specific primer (PuRV1-ORF2-4 and PuRV1-5RACE) was used for the amplification of 5′-end fragment.

The 3′-end of PuRV1 was determined by 3′-RACE. First-strand cDNA was synthesized from the total nucleic acid extracted from UOP226 using the ReverTra Ace reverse transcriptase (Toyobo, Osaka, Japan) with the oligo Tagged dT RT primer (Table S2), followed by PCR using the PrimeStar GXL DNA polymerase (Takara Bio) with a sense primer (PuV-2-fw) and the adaptor primer (Tagged-primer). The resulting PCR products were cloned into pCR-BluntII-TOPO (Invitrogen, Waltham, MA, USA) and six clones were then sequenced.

The cDNA of full-length dsRNA was synthesized by superscript VI (Invitrogen) with 5′- and 3′-UTR specific primers. The cDNA was amplified in three fragments ca. 3.0 kbp by PrimeStar GXL DNA polymerase (Takara Bio). The amplicons were directly sequenced through the Sanger method using internal primers designed from the contig. Sanger sequencing was carried out by Eurofins Genomics Co., Ltd., with ABI 3730 DNA sequencer (Applied Biosystems, Foster City, CA, USA). Obtained sequences were assembled and analyzed by DNA Dynamo software (BlueTractor Software, Gwynedd, UK).

### 2.7. Bioinformatic Analysis

The contigs obtained by high-throughput sequencing were searched using the BLAST programs on the NCBI web server for the non-redundant database (https://blast.ncbi.nlm.nih.gov/Blast.cgi, accessed on 2 July 2018, 25 January and 20 April 2021). ORFs in the contigs were predicted by DNA Dynamo software (BlueTractor Software). The slippery sequence was predicted by the FSFinder2 (http://wilab.inha.ac.kr/fsfinder2/, accessed on 11 January 2021). RNA pseudoknot structure was predicted by DotKnot (https://dotknot.csse.uwa.edu.au/, accessed on 14 January 2021) using 50 nt downstream of the predicted slippery sequence. RNA secondary structures were visualized by Pseudoviewer [32]. The conserved domains and motifs in the ORF were searched by PROSITE (https://prosite.expasy.org/, accessed on 25 January 2021) and MOTIF Search (https://www.genome.jp/tools/motif/, accessed on 25 January 2021). The RdRp domain sequences were aligned by MAFFT 7.423 [33] with an accurate option (L-INS-i). Internal ribosome entry site (IRES) structure was predicted by IRESpy (https://irespy.shinyapps.io/IRESpy/, accessed on 16 August 2021) [34].

### 2.8. Phylogenetic Analyses

Phylogenetic analysis was performed following previous studies [23,24]. Details of viral genes used for phylogenetic analysis are shown in Tables S4 and S6. Multiple sequence alignments were implemented using MAFFT 7.423 with an accurate option (L-INS-i) [33]. For the maximum likelihood (ML) phylogenetic analysis, the unreliable sites of alignment were removed by TrimAl 1.2 with the strictplus or nogap options [35]. The best-fit amino acid substitution model according to AIC was determined by ProtTest 3.4 [36]. The selected best-fit substitution model (LG + I + G + F) was used for the ML phylogenetic analysis by PhyML 3.1 [37]. The branch supports were calculated by the Simodaira–Hasegawa-like procedure approximate likelihood ratio test (aLRT SH-like) [37]. The Newick tree was visualized by the Figtree (downloaded from http://tree.bio.ed.ac.uk/software/, accessed on 16 August 2021). Branch support values larger than 0.6 are shown in the figure.

For the Bayesian analysis, the unreliable sites of alignment were removed by TrimAl 1.2 with the strictplus and nogap options. The best-fit amino acid substitution model according to BIC was determined by ProtTest 3.4. The selected best-fit substitution model (LG + I + G + F or LG + I + G) was used for the Bayesian analysis by Mrbayes 3.2.1 [38]. Two runs with four chains were run, and generations continued until the ASDSF value was below 0.01. Trees were sampled at every 100 generations. The first 25% of trees were discarded as burn-in, with the remaining trees used for generating the consensus tree. The consensus tree was visualized by the Figtree software. Bayesian posterior probability values larger than 0.5 are shown in the figure.

### 2.9. Codon Usage Bias Analysis

Coding sequences (CDSs) of *G. ultimum*, *Ph. cactorum*, *Pl. viticola*, *Saccharomyces cerevisiae* (ascomycete), and *Lentinula edodes* (basidiomycete) were obtained from Ensembl Project websites (https://protists.ensembl.org/info/data/ftp/index.html and http://fungi.ensembl.org/info/data/ftp/index.html, accessed on 16 August 2021). Because the CDS data set of *Ph. infestans* was incomplete (CDSs contained many sequences without termination codons), we used *Ph. cactorum*. Then, the cumulative Relative Synonymous Codon Usage (RSCU) of CDSs was calculated using the GCUA program [39]. The similarity index of codon usage bias among oomycetes and fungi was calculated by the D(A,B) formula [40]. When the D(A,B) value is closer to zero, the two codon usage patterns should have a higher similarity.

To analyze the similarity of codon usage bias between each fusarivirus and host, the Codon Adaptation Index (CAI) [41], the Effective Number of Codons (ENc) [42], GC3s %, and Relative Codon Deoptimization Index (RCDI) [43] were calculated by the CAIcal website (https://ppuigbo.me/programs/CAIcal/, accessed on 16 August 2021). For calculation of CAI and RCDI, a codon usage table was created on the COUSIN website (https://cousin.ird.fr/create_table.php, accessed on 16 August 2021) using CDSs obtained from Ensembl Project websites. The ENcs vs. GC3s plot with a standard curve (ENc = 2 + GC3s + 29/(GC3s^2 + (1 − GC3s)^2)) was generated.

## 3. Results

### 3.1. Detection of Viral-Like dsRNAs in the G. ultimum Isolates in Japan and Norway

**Figure 1:**
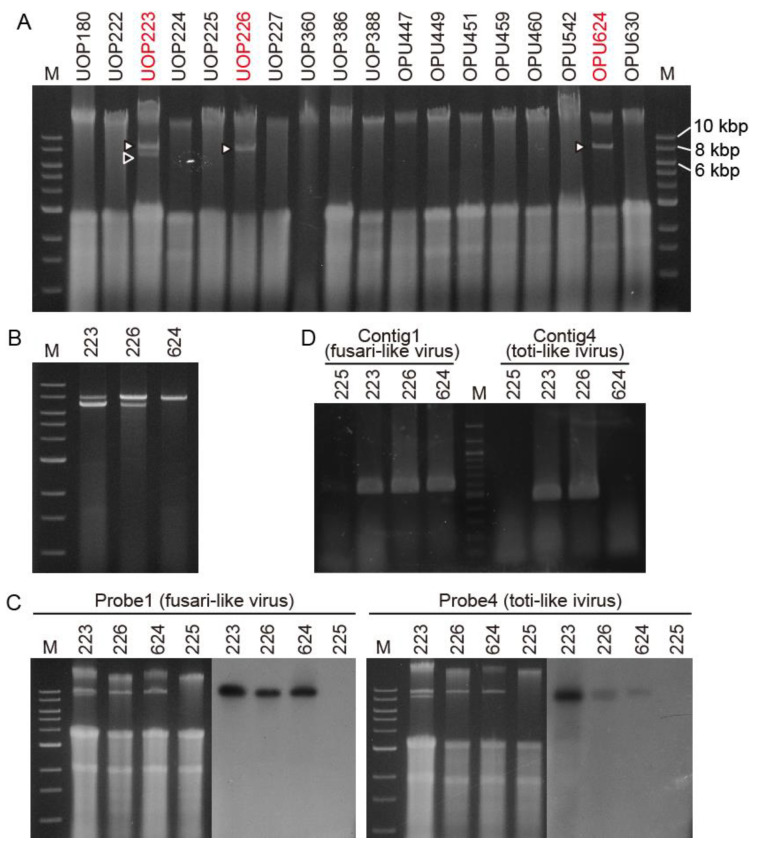
(**A**), Presence or absence of virus-like dsRNA in total nucleic acids extracted from *G. ultimum* isolates in Japan and Norway. (**B**), dsRNAs purified by CF11 cellulose column chromatography. (**C**), Northern blot analysis of fusari-like Pythium ultimum RNA virus 1 (PuRV1) and toti-like Pythium ultimum RNA virus 2 (PuRV2). dsRNAs were observed using 1% (*v*/*v*) agarose gel electrophoresis with ethidium bromide staining. M is a DNA marker (NEB, 1 kbps). (**D**), The RT-PCR analysis of fusari-like PuRV1 and toti-like PuRV2 using specific primer sets. M is a DNA marker (NEB, 100 bps).

First, we investigated the presence of viral-like dsRNA molecules in the total nucleic acid extracted from 18 isolates of *Globisporangium* and *Pythium* (Table S1). We detected nucleic acid band(s) between 6.0 and 8.0 kbp of the DNA marker in *G. ultimum* isolates UOP223, UOP226, and OPU624 in Japan (Figure 1A). After dsRNA enrichment using CF11 cellulose column chromatography, two dsRNAs of ca. 8.0 and 7.0 kbp were detected in UOP223 and UOP226, and a ca. 8.0 kbp dsRNA was detected in OPU624 (Figure 1B).

*G. ultimum* UOP226 from Hokkaido was used for further detailed analyses. The CF11-purified dsRNA treated with DNase I and S1 nuclease was used for RNA-seq with a MiSeq sequencer. After RNA-seq and de novo assembly of trimmed raw reads using the Velvet program, four large contigs of 2555, 1586, 1864, and 1031 nt, designated as contigs 1–4, were obtained. A tBLASTx analysis showed that contigs 1 and 2 were related to RNA of the RdRp of fusariviruses, and contig 4 was related to RdRp of PpRV1, a toti-like dsRNA virus from *G. polare* [24]. A tBLASTx analysis did not find similar proteins in contig 3. Dig-labeled PCR probes for contigs 1 and 4 (probe 1 and probe 4) were prepared for Northern blot analysis. Probe 1 annealed with the ca. 8.0 kbp dsRNA while probe 4 annealed with the ca. 7.0 kbp dsRNA (Figure 1C). Because a mild signal for probe 4 was observed in OPU624 by the Northern blot analysis, we performed RT-PCR with specific primers for contigs 1 and 4 to confirm the viral infection. The contig 1 primer set amplified the target bands in all three isolates, while the contig 4 primer set detected the target bands in UOP223 and UOP226 (Figure 1D), confirming that OPU624 was not infected with the toti-like virus. We named the ca. 8.0 and ca. 7.0 kbp dsRNA viruses Pythium ultimum RNA virus 1 (PuRV1) and Pythium ultimum RNA virus 2 (PuRV2), respectively.

### 3.2. Pythium ultimum RNA Virus 1 (PuRV1)

#### 3.2.1. Genome Sequencing of PuRV1

The trimmed-reads from the MiSeq analysis were de novo assembled using the rnaSPAdes program, and three contigs with more than 1000 nt were obtained (1501, 4519, and 5307 nt). The sequences of three contigs were identical and showed homology to contigs 1 and 2 related to fusarivirus. We also found that contig 3 was assembled with this 5307 nt long contig. Therefore, we combined the contigs acquired from the Velvet and rnaSPAdes programs and obtained a 6686 nt sequence of PuRV1. Forward and reverse primers were designed in the 6686 nt contig (Table S2), and a 6513 nt sequence was confirmed by RT-PCR and direct sequencing of the amplified products, identifying 18 base wrong insertions in the ORF of the 6686 nt sequence obtained by MiSeq analysis. In addition, RT-PCR using dT primers for the poly-A tail, which most fusariviruses are known to possess, amplified the target sequence of PuRV1, indicating that the PuRV1 genome had the poly-A tail of the 3′-end. Therefore, the 3′-end sequence of PuRV1 was determined by the 3′-RACE using the oligo dT-adaptor primer in RT reaction. The RLM-RACE also determined the 5′-end sequence of PuRV1. After correction of 18 base wrong insertions and of the 5′- and 3′-ends sequences, the 6686 nt sequence with 25 nt poly A of PuRV1 genome was finally deposited in the NCBI database with the accession number LC622068.

#### 3.2.2. Organization of PuRV1 Genome

**Figure 2:**
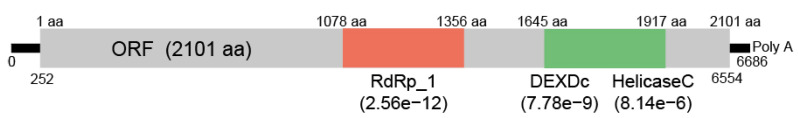
The genome structure of PuRV1. The boxed region shows an open reading frame (ORF). The red and green colored boxes show predicted RdRp_1 and Helicase domains, respectively.

A large open reading frame (ORF) (6303 nt, 2101 aa) was found in the PuRV1 6686 nt genome with a CG content of 42%. Two single-nucleotide polymorphisms (SNPs) were found in the ORF by both MiSeq and Sanger sequencing. For nt 1519, 1891 of 2184 reads (88%) were A, and 253 of 2184 reads (12%) were G. For nt 5600, 628 of 677 reads (92%) were C, and 47 of 677 (8%) reads were U. The nt 5600 SNP was a synonymous substitution, while the nt 1519 SNP was a non-synonymous substitution {GAT(D) and GGT(G)}. A tBLASTx analysis showed that the RdRp domain (pfam00680, *E*-value = 2.56 × 10^−12^) and the Hel domains {DEXDc (smart00487 E-value = 7.78 × 10^−9^) and Helicase_C (pfam00271 *E*-value = 8.14 × 10^−6^)}, were conserved in the ORF (Figure 2). In addition, the ORF had sequence similarity to the RdRps of fusariviruses (Table S3). The top three hits were PvlaFV2 (identity, 31.3%; query cover, 50%; *E*-value = 1.00e−135), Lentinula edodes fusarivirus 2 (LeFV2) (identity, 32.3%; query cover, 43%; *E*-value = 2.00 × 10^−135^), and Penicillium roqueforti ssRNA mycovirus 1 (PRRMV1) (identity, 31.7%; query cover, 49%; *E*-value = 4.00 × 10^−135^).

#### 3.2.3. Phylogenetic Analysis of PuRV1

**Figure 3:**
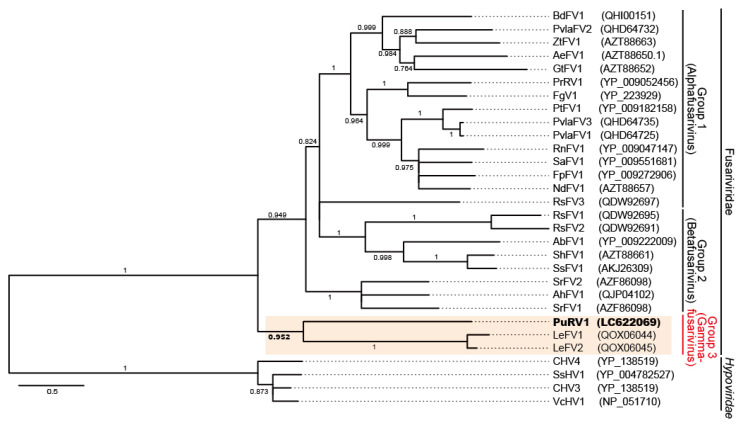
The maximum likelihood tree (LG + I + G + F) based on amino acids of RdRp from fusariviruses. RdRps of hypoviruses (CHV3, CHV4, SsHV1, and VcHV1) were used for rooting. The numbers on branches indicate the results of SH-like approximate likelihood ratio tests (aLRTs). Branch support values larger than 0.6 are shown. Scale bar shows substitutions per site.

A maximum likelihood (ML) tree (LG + I + G + F), rooted by hypoviruses (CHV4, SsHV1, CHV3, and VcHV1), was constructed from the 928 sites of the deduced RdRp amino acid sequence of PuRV1 and those of 26 fusariviruses (Table S4). PuRV1 represented a monophyletic group with LeFV1 and LeFV2 from shiitake mushroom *L. edodes* (basidiomycete) [44] with a high aLRT value = 0.952 (Figure 3). Even if the highest homology of the full-length ORF of PuRV1 was found with PvlaFV2, PuRV1 belonged to a different phylogenetic group from PvlaFV1-3, which was detected from the grapevine downy mildew *Pl. viticola*, when a phylogenetic tree was constructed using the RdRp-helicase region in the ORF. The same conclusion was obtained using Bayesian analysis (Figure S1).

### 3.3. Pythium ultimum RNA Virus 2 (PuRV2)

#### 3.3.1. Genome Sequencing of PuRV2

Because we did not obtain a long contig similar to totiviruses when using the rnaSPAdes program and data from the MiSeq analysis, we conducted high-throughput sequencing analysis with DNBSEQ-G400. After assembling the short row reads using the rnaSPAdes program, a 5867 nt long contig, homologous to the above toti-like contig 4, was obtained. By using the forward and reverse primers designed in the 5867 nt long contig (Table S2), a nearly complete PuRV2 genome was confirmed by RT-PCR and direct sequencing. The RLM-RACE also determined the 5′- and 3′-ends sequences of PuRV2. Finally, the complete sequence of PuRV2 was determined to be 5864 nt with a GC content of 59% and was deposited in the NCBI database with the accession number LC622069.

#### 3.3.2. Organization of PuRV2 Genome

**Figure 4:**
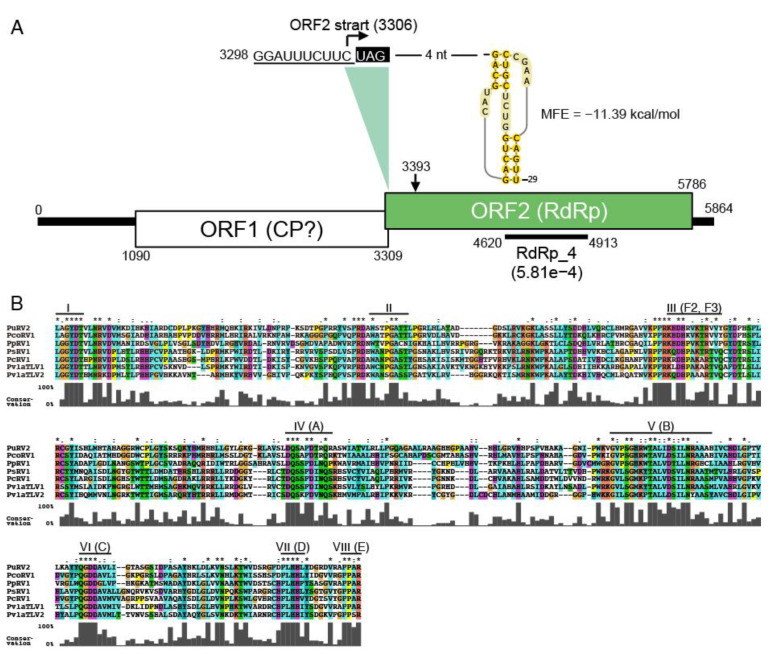
(**A**), The genome structure of PuRV2. The underlined sequence shows putative slippage sites and putative H-type pseudoknot to the right. Boxed regions show two open reading frames (ORFs). (**B**), Comparison of the amino acid sequence of the conserved RdRp region of PuRV2 and the other toti-like viruses from oomycetes. Amino acid sequences were aligned using MAFFT 7.423. I-IV(E) indicate the eight conserved motifs in the RdRps of dsRNA viruses [45,46,47]. Consensus symbols were assigned according to the BLOSUM62 scoring matrix: *, identical; nonidentical with score ≥1; nonidentical with score = 0.

The PuRV2 genome contained two overlapping large ORFs in different frames (Figure 4A). PuRV2 had a 1089 nt long 5′-untranslated region (UTR) that contained 15 AUG codons upstream from the first AUG codon in ORF1. The 5′-UTR included six small ORFs of 20 amino acids or more. An IRES structure was not predicted in 5′-UTR by the IRESpy program. The slippery-like sequence “GGAUUUCUUUC” and H-type RNA pseudoknot with maximum free energy of −11.39 kcal/mol were predicted near the stop codon of ORF1 (Figure 4A).

The ORF1 (1090–3309 nt) encoding protein with 740 aa was similar to ORF1 of PcoRV1 (QTT60990.1; ident, 51.6%; Query cover, 97%; *E*-value = 0.0) and PpRV1 (YP_009552274.1; ident, 27.2%; Query cover, 52%; *E*-value = 9.00e−13). ORF2, which overlapped in the -1 frame with ORF1, was predicted to begin with C at 3306 nt in the slippery-like sequence, while the first methionine codon (3393–3395 nt) was located at aa 30 (Figure 4A). ORF2 (3306–5786 nt), encoding a protein with 827 aa, was similar to RdRps of toti-like viruses identified from oomycetes (Table 1 and Table S5). The RdRp_4 domain (439–536 aa, E-value 5.8E-4) and the eight conserved motifs of the RdRp of dsRNA viruses [45,46,47] were found in the ORF2 (Figure 4A,B). The amino acid and nucleotide sequence identity of the RdRp_4 domain among seven toti-like oomycete viruses was analyzed using the BLAST program. Table 2 shows the percent identity when the query coverage was more than 50%. In the conserved RdRp_4 domain, the amino acid percent identity ranged from 33.9 to 65.9% (Table 2). Significant nucleotide identity was found only in two cases: 65.0% between PuRV2 and PcoRV1 and 68.0% between PsRV1 and PcRV1 (Table 2).

#### 3.3.3. Phylogenetic Analysis of PuRV2

**Figure 5:**
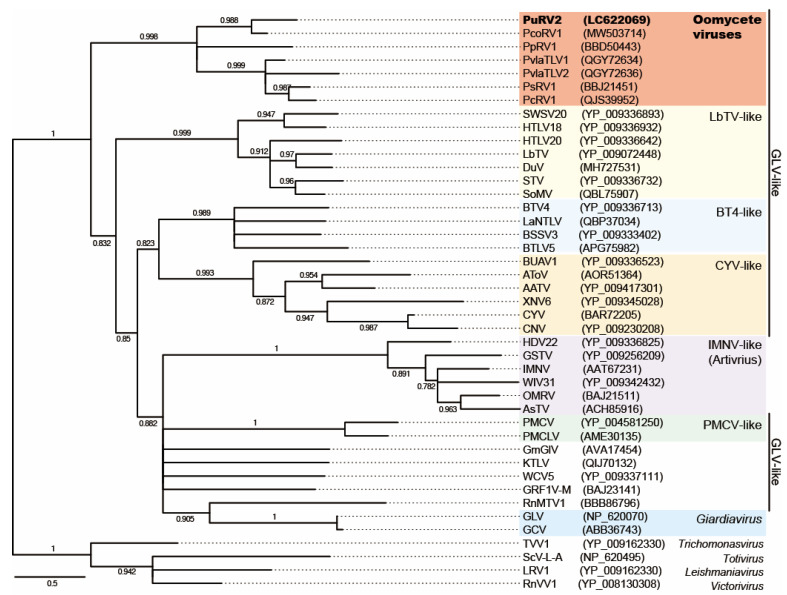
The maximum likelihood tree (LG + I + G + F) based on amino acids of the RdRp domain from viruses in the GLV-like and IMNV-like groups. RdRp of *Totivirus* (ScV-L-A), *Victorivirus* (RnVV1), *Trichomonasvirus* (TVV1), and *Leishmaniavirus* (LRV1) in the family *Totiviridae* were used for rooting. The numbers on branches indicate the results of SH-like approximate likelihood ratio tests (aLRTs). Branch support values larger than 0.6 are shown. Scale bar shows substitutions per site.

Toti-like oomycete viruses have a phylogenetic relationship with the arthropod GLV-like viruses in the family *Totiviridae* [8,23,24]. Thus, a ML tree (LG + I + G + F), rooted by *Totivirus* (ScV-L-A), *Victorivirus* (RnVV1), *Trichomonasvirus* (TVV1), and *Leishmaniavirus* (LRV1) as references, was constructed from the 194 sites of deduced amino acid sequences of the conserved RdRp domain in the ORF2 of PuRV2, PpRV1, PsRV1, PcoRV1, PcRV1, PvlaTLV1, and PvlaTLV2, which were isolated from oomycetes, and those of 32 confirmed and putative GLV- and IMNV-like viruses from family *Totiviridae* [23,48] (Figure 5, Table S6). As expected, PuRV2 represented a monophyletic group with PpRV1, PsRV1, PcoRV1, PcRV1, PvlaTLV1, and PvlaTLV2 with a high aLRT value = 0.998. The same conclusion was obtained using Bayesian analysis (Figure S2). Phylogenetic trees based on the amino acid sequences of ORF1 and ORF2 of the seven toti-like oomycete viruses (PuRV2, PpRV1, PsRV1, PcoRV1, PcRV1, PvlaTLV1, and PvlaTLV2) were also constructed using the ML method (Figure S3). The phylogenetic tree did not differ between ORF1 and ORF2, and toti-like viruses from *Globisporangium* and *Phytophthora* were mixed within a single phylogenetic group.

### 3.4. Codon Usage Bias Analysis of PuRV1

Tian et al. indicated that the narrow host spectrum positive-sense ssRNA viruses have a similar overall codon usage pattern to their hosts [49]. Fusariviruses have been found in fungi and oomycetes only [21,50,51,52,53], suggesting the possibility that the codon usage bias of fusariviruses may be adapted to those of their host. In addition, one of the characteristics of the PuRV1 genome is the AT-rich sequence (AT 58%). Therefore, we compared the codon usage bias of the RdRp genes of fusariviruses to those of oomycetes (*G. ultimum*, *Ph. cactorum*, and *Pl. viticola)* and fungi (*S. cerevisiae*, and *L. edodes)*. First, we compared the codon usage bias among oomycetes and fungi. The RSCU was calculated using CDSs obtained from Ensembl Project websites, and the similarity index D(A,B) values among oomycetes and fungi host were calculated (Tables S7 and S8). When the D(A,B) value is closer to zero, the two codon usage patterns should have higher similarity [40]. The D(A,B) value between *G. ultimum* and *Ph. cactorum* was 0.01347 (Table S8), indicating that the codon usage biases of *G. ultimum* and *Ph. cactorum* were most similar. The codon usage bias of *Pl. viticola* was slightly different from those of *G. ultimum* and *Ph. cactorum* {(D(A,B) value = 0.07715 and = 0.04284, respectively}, while the codon usage bias of *Pl. viticola* was most similar to *L. edodes* {D(A,B) value = 0.01755} (Table S8). Thus, *Pl. viticola* showed differences in codon usage bias among three plant-parasitic oomycetes.

**Figure 6:**
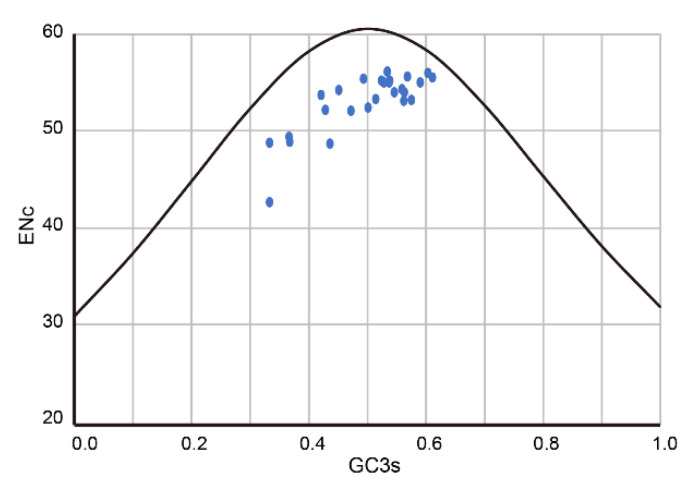
ENc values plotted against GC3s. The blue circles represent individual fusariviruses.

Next, we calculated the CAI of RdRps of PuRV1 and the other fusariviruses for those of the oomycetes and fungi (Table 3). The CAI value will generally be higher if the virus uses the preferred codons of the host’s genes. The CAI values of the PuRV1 ORF were 0.522, 0.657, 0.819, 0.761, and 0.855 for *G. ultimum*, *Ph. cactorum*, *Pl. viticola*, *S. cerevisiae*, and *L. edodes*, respectively. The CAI values of the RdRp coding ORF of PvlaFV1 from the *Pl. viticola* were 0.528, 0.664, 0.772, 0.727, and 0.848 for *G. ultimum*, *Ph. cactorum*, *Pl. viticola*, *S. cerevisiae*, and *L. edodes*, respectively. The CAI values of the LeFV2 ORF from *L. edodes* were 0.469, 0.623, 0.802, 0.786, and 0.866 for *G. ultimum*, *Ph. cactorum*, *Pl. viticola*, *S. cerevisiae*, and *L. edodes*, respectively. The CAI values of other fusariviruses showed a similar trend (Table 3), indicating that the RdRp gene of fusariviruses has a codon usage bias more similar to those of *Pl. viticola*, *S. cerevisiae,* and *L. edodes* than *G. ultimum* and *Ph. cactorum*. We also performed the RCDI analysis to quantify the cumulative effect of a particular codon bias in a protein-coding sequence. RCDI values equal to 1 indicate that the virus has a host-adapted codon usage pattern. In contrast, RCDI values >1 indicate less adaptability. For comparison, the RCDI values of the PuRV1 and LeFV2 ORFs were higher for *G. ultimum* and *Ph. cactorum* than for *Pl. viticola*, *S. cerevisiae*, and *L. edodes* (Table 4). These results showed that the PuRV1 ORF had a codon usage bias more adapted to fungi than *Globisporangium* and *Phytophthora*. In contrast, the RdRp ORF of PvlaFVs had a codon usage bias similar to their host *Pl. viticola*. An ENc-GC3s plot analysis was performed to assess the forces influencing the codon usage bias of the RdRp gene of fusariviruses. In general, points below the expected ENc curve indicate that the codon usage is affected by natural selection rather than mutational pressure [54]. All fusariviruses were clustered below the expected ENc curve (Figure 6), indicating that codon usage of fusarivirus RdRp was influenced by natural selection.

## 4. Discussion

This study discovered unclassified non-segmented fusari- and toti-like viruses from the *G. ultimum*, named PuRV1 and PuRV2, respectively. To our knowledge, this is the first report of viruses identified from *G. ultimum*. In the Northern blot analysis, PuRV1 was detected in three *G. ultimum* isolates, and PuRV2 was detected in two *G. ultimum* isolates in Japan (Figure 1C), indicating that the same viruses infected *G. ultimum* isolates from different regions of Japan. PuRV1 has a large ORF that encodes the RdRp of ssRNA viruses (Figure 2). Molecular phylogenetic analysis of the RdRp domain amino acid sequence of fusariviruses showed that PuRV1 shared a phylogenetic lineage with LeFV1 and LeFV2 from fungus *L. edodes* [44], but not with PvlaFVs from oomycete downy mildew (Figure 3). Interestingly, the codon usage bias of PuRV1 ORF was more adapted to those of fungi than *Globisporangium* and *Phytophthora* (Table 3 and Table 4). PuRV2 has two large ORFs, and ORF2 encodes the RdRp related to toti-like oomycete viruses (Figure 4). Molecular phylogenetic analysis of the RdRp domain amino acid sequence of GLV- and IMNV-like viruses showed that PuRV2 represented a monophyletic group with other toti-like oomycete viruses (Figure 5). However, significant nucleotide similarity was not found in most combinations among toti-like oomycete viruses (Table 2).

### 4.1. Pythium ultimum RNA Virus 1 (PuRV1)

Fusariviruses, found in fungi and oomycetes, are plus- non-segmented ssRNA viruses [21,50,51,52,53]. Fusariviruses have a poly-A tail in the 3′-end of the viral genome [55]. Most fusariviruses have multiple ORFs, with ORF1 encoding the RdRp of ssRNA viruses [50,51,52,53]. Although there is no threshold for determining the species identity of fusariviruses, Gilbert et al. recommended two criteria to qualify viruses as new fusarivirus species: a unique fungal host and ≤60% identity of the RdRp sequence in common with existing fungal sequences [51]. PuRV1 shared only ~30% amino acid sequence identity with LeFV1 and LeFV2, suggesting that PuRV1 is a novel species of fusarivirus.

Previous molecular phylogenetic studies indicated that the fusariviruses could be divided into two groups [47,48,49]. Group 1 fusariviruses have structural maintenance of chromosome (SMC)-like or Spc7 domains, while almost all group 2 fusariviruses, except MiFV1, lack these domains [47,48]. Honda et al. also reported that the group 1 fusariviruses have the coiled-coil domain on ORF2, but group 2 fusariviruses do not [49]. Recently, Guo et al. discovered fusari-like viruses from the shiitake mushroom *L. edodes* (LeFV1-3) [53]. LeFVs and PuRV1 were divided from groups 1 and 2 fusariviruses (Figure 3) and had the following characteristics in common; (1) only one ORF was found in the viral genome, and (2) no SMC-like and coiled-coil domains were found in an ORF. Based on the molecular phylogenetic analysis and the viral genome characters, PuRV1 and LeFVs are distinct from group 1 and 2 fusariviruses. During the review of this manuscript, a proposal was submitted to the ICTV to approve a new family, *Fusariviridae* (https://talk.ictvonline.org/files/proposals/taxonomy_proposals_fungal1/m/fung04, accessed on 16 August 2021). In this proposal, viruses in the family *Fusariviridae* will be divided into three genera, and LeFVs will be classified as genus *Gammafusarivirus*. Thus, according to this proposal, PuRV1 may be classified as a new species of *Gammafusarivirus* together with LeFVs.

### 4.2. Pythium ultimum RNA Virus 2

Totiviruses have been discovered in excavata, alveolate, fungi, arthropods, fish, and oomycetes [24,48,56]. The family *Totiviridae* contains five genera: *Totivirus*, *Giardiavirus*, *Victorivirus*, *Leishmaniavirus*, and *Trichomonasvirus* (ICTV, Virus Taxonomy: 2020 Release) and there are many unclassified toti-like viruses. As expected, PuRV2 from *G. ultimum* represented a monophyletic group with the other toti-like oomycete viruses PpRV1, PsRV1, PcoRV1, PcRV1, PvlaTLV1, and PvlaTLV2 in the clade containing the genus *Giardiavirus*, GLV-like viruses, and IMNV-like viruses (Figure 5). Most viruses in this clade are arthropod viruses, and some of them are protozoan, fungus, and fish viruses [24]. The toti-like oomycete viruses have similar characters; (1) full-length viral genomes are around 5500 nt, (2) long 5′-UTR are more than 700 nt, (3) the 5′-UTR contains some AUG codons upstream from the first AUG codon in ORF1, (4) a slippery sequence and RNA pseudoknot near the stop codon of ORF1 causes -1 ribosomal frameshift and translation of ORF1 and ORF2 fusion protein (CP-RdRp fusion). In particular, the long 5′-UTR is a unique character of toti-like oomycete viruses that distinguishes them from other GLV-like viruses. Because the amino acid sequence identities of the ORF1 (CP) and the ORF2 (RdRp) between PuRV1 and PcoRV1 are 51.6% and 60.6%, respectively, we propose the PuRV1 as a novel virus species in the family *Totiviridae*.

### 4.3. Origins of PuRV1 and PuRV2

Our findings allow us to discuss the different origins of the fusari-like PuRV1 and toti-like PuRV2 co-infecting *G. ultimum*. Phylogenetic analyses have revealed that plant-parasitic oomycetes, including *Globisporangium*, *Pythium*, *Phytophthora*, and downy mildew, appear to have radiated from a common plant-parasitic ancestor [1,2]. The deduced amino acid sequences of the RdRp domain from toti-like oomycete viruses showed a single phylogenetic group with a similar genomic structure (Figure 5). In addition, the deduced RdRp amino acid sequence of toti-like viruses discovered in diatom *Thalassiothrix antarctica* and the transcriptome shotgun assembly sequence of a diatom *Nitzschia* sp. were phylogenetically grouped with toti-like oomycete viruses [23,24,57]. Oomycetes are phylogenetically related to diatoms and brown algae in the stramenopiles [1]. Thus, it is reasonable to consider that the common ancestor of toti-like oomycete viruses infected the stramenopile ancestor of these oomycetes and coevolved with the host oomycete. However, only the conserved domain of RdRp of toti-like oomycete viruses shares moderate identity on the amino acid level and virtually no similarity on the nucleotide level, except for two combinations (Table 2). In addition, phylogenetic trees based on the deduced amino acid sequences of ORF1 and ORF2 of the toti-like oomycete viruses showed that toti-like viruses from *Globisporangium* and *Phytophthora* were mixed within a single phylogenetic group (Figure 5 and Figure S3). Thus, the nucleotide sequence of toti-like oomycete viruses is not so homologous, and the phylogenetic relationship of the host *Globisporangium*, *Phytophthora*, and downy mildew does not match the phylogenetic tree of the toti-like oomycete viruses, suggesting the possibility of convergent evolution of toti-like oomycete viruses. The different ancestral toti-like viruses might have infected each host oomycete independently, and convergent amino acid evolution to adapt the oomycetes resulted in the amino acid similarity in the RdRp region of each toti-like oomycete virus. Indeed, convergent evolution of the coat proteins of totivirus and the RdRps of dsRNA viruses was previously considered in several papers [58,59,60,61].

In contrast to toti-like oomycete viruses, fusari-like PuRV1 from *G. ultimum* belongs to a different phylogenetic group from PvlaFVs from *Pl. viticola*. These fusari-like oomycete viruses had different viral genome structures; PuRV1 has one ORF, but PvlaFVs encode three ORFs. Interestingly, the codon usage bias of PuRV1 is more similar to that of fungi than *Globisporangium* and *Phytophthora* (Table 3 and Table 4). For RNA viruses, RNA structure of the viral genome is most likely more important than codon usage bias [62,63,64,65]. However, Tian et al. reported that the narrow host spectrum positive-sense ssRNA viruses might have a similar overall codon usage pattern to their hosts [49]. Because most fusariviruses have been discovered from fungi, our CAI and RCDI analyses suggest that the codon usage bias of fusariviruses is adapted to the fungal host. Recently cross-kingdom horizontal transmission of viruses between plants and fungi has been suggested (reviewed in [66]). In nature, plants co-exist with diverse microbes such as archaea, bacteria, fungi, and oomycetes. These microorganisms interact with each other within the plant holobiont [67]. The horizontal gene transfer from fungi to oomycetes has previously been suggested [68]. Co-occurrence of plant-parasitic fungi and oomycetes on the roots and leaves of the colonized plants have been reported [27,69,70,71]. Because PuRV1 and its closely related LeFVs have codon usage bias adapted to fungi, we hypothesized that the PuRV1 ancestor, which was different from the PvlaFV ancestor, might have been horizontally transmitted from fungi to the *G. ultimum* ancestor. In the genome analysis of PuRV1, we found that one SNP (5600 nt) was a synonymous substitution; the codon containing 5600 nt encodes Asp, and the percentages of synonymous codons were GAC (88%) and GAU (12%). The RSCU values of these synonymous codons for G. *ultimum* (GAC = 1.31 and GAU = 0.69) and *L. edodes* (GAC = 0.89 and GAU = 1.11) suggest that the PuRV1 prefers the GAC codon that is the most frequently used by *G. ultimum* (Table S7). Although it is only one SNP, this may represent the adaptation process of the codon usage bias of PuRV1 to *G. ultimum*.

## Figures and Tables

**Table 1:** List of oomycete viruses that were hit by the tBLASTx search of the PuRV2 ORF2.

Description	Query Cover	E-Value	Per. Ident	Accession
RNA-dependent RNA polymerase (Phytophthora condilina RNA virus 1)	92%	0.0	60.6	QTT60989.1
RNA-dependent RNA polymerase (Pythium polare RNA virus 1)	88%	9.00 × 10^−87^	34.9	YP_009552275.1
Putative RNA-dependent RNA polymerase (Plasmopara viticola lesion-associated totivirus-like 1)	63%	1.00 × 10^−67^	34.1	QGY72634.1
Putative RNA-dependent RNA polymerase (Plasmopara viticola lesion-associated totivirus-like 2)	58%	1.00 × 10^−64^	34.9	QGY72636.1
CP-RdRp fusion protein (Phytophthora cactorum RNA virus 1)	62%	5.00 × 10^−61^	33.4	QJS39952.1
CP-RdRp fusion protein (Pythium splendens RNA virus 1)	63%	8.00 × 10^−55^	32.8	BBJ21453.1
CP-RdRp fusion protein (Pythium splendens RNA virus 1)	63%	1.00 × 10^−54^	32.8	BBJ21451.1

**Table 2:** Amino acid (white cells) and nucleotide (gray cells) sequence identities of RdRp_4 domain among toti-like oomycete viruses.

	PuRV2	PpRV1	PsRV1	PcRV1	PcoRV1	PvlaTLV1	PvlaTLV2
PuRV2		41.5	35.2	35.3	63.9	36.8	35.4
PpRV1	no ^a^		37.8	38.4	41.2	39.0	33.9
PsRV1	no	no		65.9	36.2	59.9	48.3
PcRV1	no	no	68.0		35.5	57.0	49.0
PcoRV1	65.0	no	no	no		38.8	38.1
PvlaTLV1	no	no	no	no	no		49.4
PvlaTLV2	no	no	no	no	no	no	

^a^ no indicates that significant similarity was not found between viruses.

**Table 3:** Codon Adaptation Index (CAI), GC contents (GC%), and Effective Number of Codons (ENc) of the RdRp gene of fusariviruses.

Virus ^a^	Length (nt)	Codon Adaptation Index (CAI)					GC%	ENc ^b^
		*Globisporangium ultimum*	*Phytophthora cactorum*	*Plasmopara viticola*	*Saccharomyces cerevisiae*	*Lentinula edodes*		
PuRV1	6303	0.522	0.657	0.816	0.761	0.855	42.9	48.7
LeFV1	5616	0.455	0.614	0.826	0.813	0.874	39.6	42.7
LeFV2	3591	0.469	0.623	0.802	0.786	0.866	41.5	48.9
PvlaFV1	4650	0.528	0.664	0.772	0.727	0.848	46.0	53.3
PvlaFV2	4614	0.550	0.687	0.756	0.697	0.843	50.8	54.0
PvlaFV3	4509	0.508	0.646	0.776	0.742	0.869	45.2	52.1
AeFV1	4671	0.575	0.707	0.750	0.678	0.823	52.2	55.5
SaFV1	4584	0.534	0.671	0.754	0.707	0.832	49.2	54.0
NdFv1	4581	0.554	0.686	0.761	0.705	0.842	49.0	55.6
FpFV1	4506	0.553	0.692	0.764	0.707	0.842	49.1	54.3
RnFV1	4629	0.560	0.691	0.788	0.722	0.851	46.6	55.0
PtFV1	4647	0.532	0.668	0.789	0.738	0.852	45.3	55.4
ZtFV1	4476	0.573	0.698	0.758	0.688	0.835	51.0	55.0
GtFV1	4578	0.542	0.683	0.771	0.710	0.848	49.1	55.0
BdFV1	4635	0.542	0.674	0.792	0.740	0.860	46.3	52.4
PrRV1	4572	0.558	0.684	0.768	0.697	0.851	49.5	53.1
FgV1	4653	0.571	0.705	0.760	0.696	0.848	51.4	53.2
SrFV1	4914	0.513	0.657	0.797	0.742	0.856	45.4	54.2
AhFV1	4914	0.578	0.702	0.741	0.668	0.823	52.3	56.0
SrFV2	5037	0.539	0.675	0.760	0.699	0.836	49.0	55.2
RsFV3	5388	0.537	0.675	0.766	0.711	0.849	47.6	55.2
AbFV1	4569	0.555	0.682	0.773	0.713	0.841	47.1	56.1
ShFV1	5157	0.446	0.583	0.798	0.801	0.853	36.3	48.8
SsFV1	4998	0.472	0.604	0.812	0.787	0.856	37.7	49.4
RsFV1	4683	0.499	0.632	0.796	0.757	0.850	41.9	52.2
RsFV2	4587	0.493	0.625	0.793	0.764	0.853	40.7	53.7

^a^ The viral full names are shown in Table S4. ^b^ The Effective Number of Codons is a general measure of codon usage bias from equal codon usage in a gene.

**Table 4:** Relative Codon Deoptimization Index (RCDI) of RdRp of fusariviruses.

Virus ^a^	Relative Codon Deoptimization Index (RCDI)				
	*Globisporangium ultimum*	*Phytophthora cactorum*	*Plasmopara viticola*	*Saccharomyces cerevisiae*	*Lentinula edodes*
PuRV1	1.935	1.549	1.269	1.127	1.233
LeFV1	2.520	1.852	1.316	1.205	1.333
LeFV2	2.222	1.655	1.252	1.140	1.204
PvlaFV1	1.809	1.395	1.325	1.172	1.148
PvlaFV2	1.521	1.252	1.282	1.332	1.139
PvlaFV3	1.888	1.461	1.316	1.220	1.133
PtFV1	1.567	1.273	1.143	1.143	1.089
ZtFV1	1.361	1.188	1.255	1.330	1.147

^a^ The viral full names are shown in Table S4.

## Data Availability

Data supporting reported results are available from GenBank under accession numbers LC622068 and LC622069.

## Supplementary Materials

The following are available online at https://www.mdpi.com/article/10.3390/v13101931/s1, Figure S1: The Bayesian tree (LG + I + G + F) based on amino acids of RdRp of fusariviruses and hypoviruses, Figure S2: The Bayesian tree (LG + I + G) based on amino acids of RdRp of *Totivirus* (ScV-L-A), *Victorivirus* (RnVV1), and viruses in the GLV- and IMNV-like groups in the family *Totiviridae*, Table S1: Isolates of *Globisporangium ultimum* used in this study, Table S2: Primers used in this study, Table S3: Hits resulting from the tBLASTx search of the PuRV1 ORF, Table S4: List of viruses used for molecular phylogenetic analysis of PuRV1, Table S5: Hits resulting from the tBLASTx search of the PuRV2 ORF2, Table S6: List of viruses used for molecular phylogenetic analysis of PuRV2, Table S7: Relative synonymous codon usage (RSCU) of oomycetes and fungi genes, Table S8: The similarity index D(A,B) value of codon usage bias among oomycetes and fungi.
