# Supplementary figures and images for: Novel Fusari- and Toti-like Viruses, with Probable Different Origins, in the Plant Pathogenic Oomycete Globisporangium ultimum
Source: Viruses. 2021 Sep 25;13(10):1931. doi: 10.3390/v13101931 (PMC8538416; doi:10.3390/v13101931)

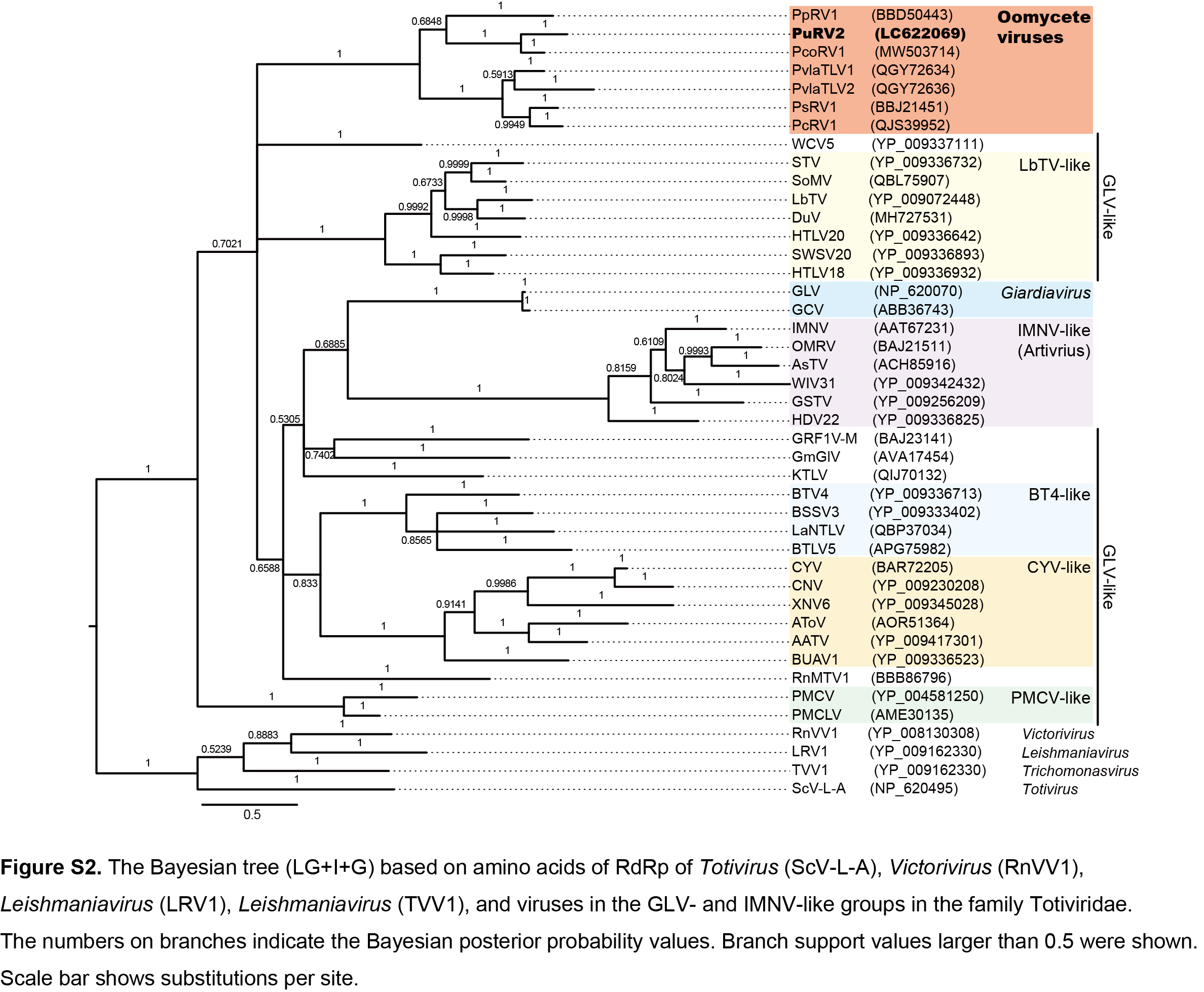

Supplement: Supplementary file 1 [file viruses-13-01931-s001.zip › supplementary files final/FigS2F.jpg]

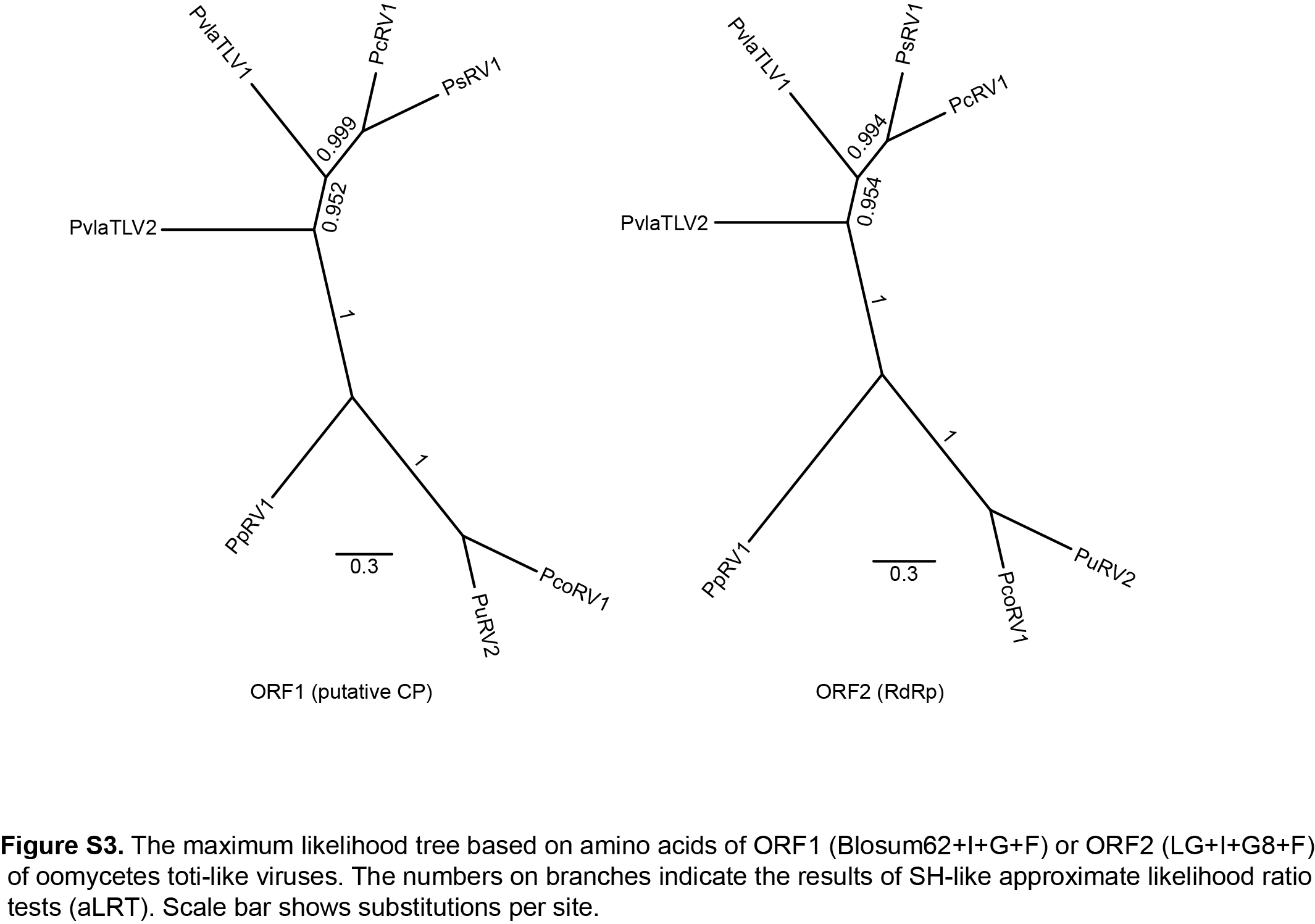

Supplement: Supplementary file 1 [file viruses-13-01931-s001.zip › supplementary files final/FigS3F.jpg]

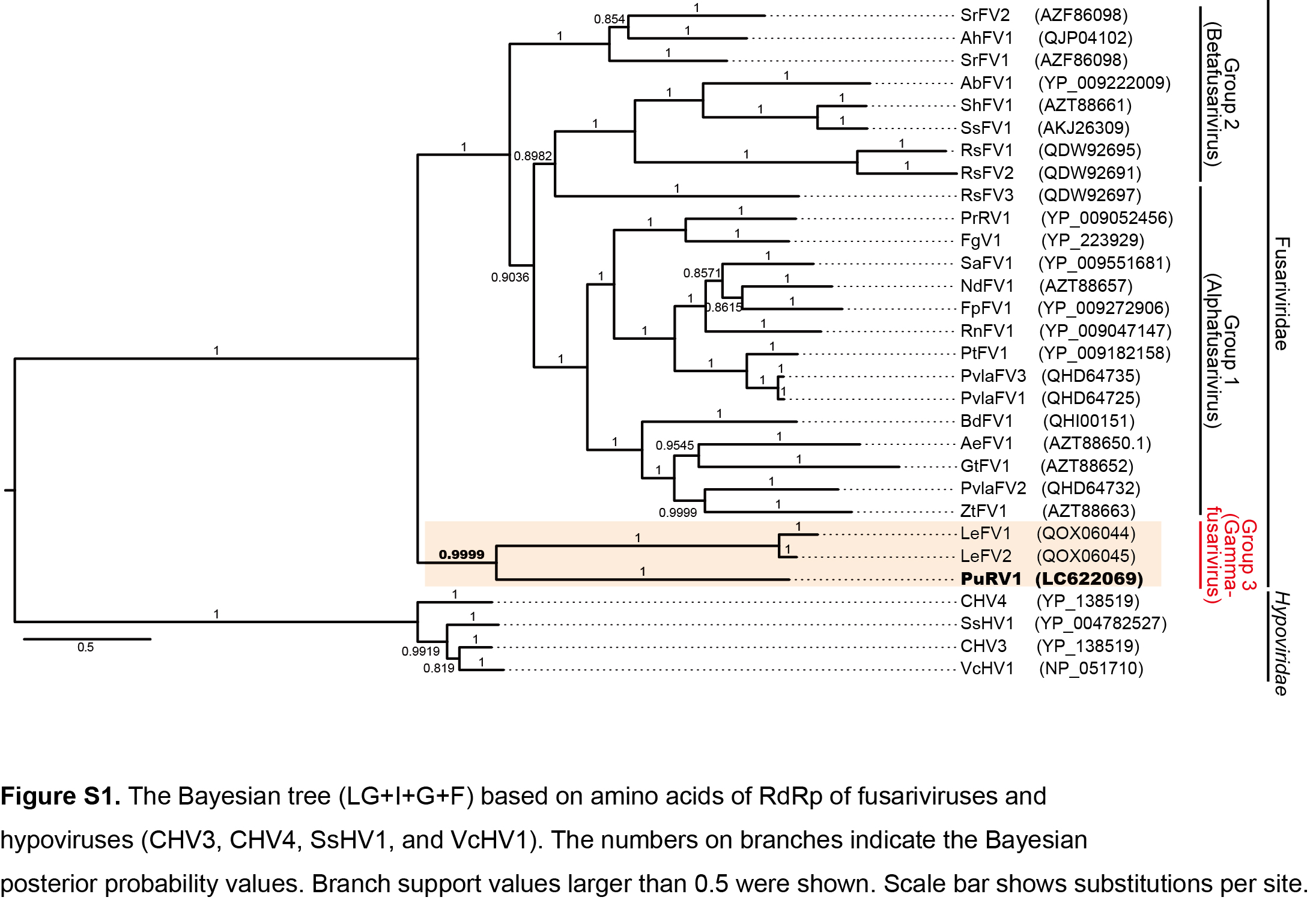

Supplement: Supplementary file 1 [file viruses-13-01931-s001.zip › supplementary files final/FigS1F.jpg]
